# Methacholine bronchial provocation measured by spirometry versus wheeze detection in preschool children

**DOI:** 10.1186/1471-2431-5-19

**Published:** 2005-06-28

**Authors:** Lea Bentur, Raphael Beck, Nael Elias, Asher Barak, Ori Efrati, Yaacov Yahav, Daphna Vilozni

**Affiliations:** 1 Pediatric Pulmonary Unit, Meyer Children's Hospital, Rambam Medical Center, and the Rappaport Faculty of Medicine, Technion – Israel Institute of Technology, Haifa, Israel; 2 Pediatric Pulmonary Unit, The Edmond and Lili Safra Children's Hospital, Chaim Sheba Medical Center, Tel-HaShomer, Ramat-Gan, Israel

## Abstract

**Background:**

Determination of PC_20_-FEV_1_ during Methacholine bronchial provocation test (MCT) is considered to be impossible in preschool children, as it requires repetitive spirometry sets. The aim of this study was to assess the feasibility of determining PC_20_-FEV_1_ in preschool age children and compares the results to the wheeze detection (PCW) method.

**Methods:**

55 preschool children (ages 2.8–6.4 years) with recurrent respiratory symptoms were recruited. Baseline spirometry and MCT were performed according to ATS/ERS guidelines and the following parameters were determined at baseline and after each inhalation: spirometry-indices, lung auscultation at tidal breathing, oxygen saturation, respiratory and heart rate. Comparison between PCW and PC_20_-FEV_1_ and clinical parameters at these end-points was done by paired Student's t-tests.

**Results and discussion:**

Thirty-six of 55 children (65.4%) successfully performed spirometry-sets up to the point of PCW. PC_20_-FEV_1_ occurred at a mean concentration of 1.70+/-2.01 while PCW occurred at a mean concentration of 4.37+/-3.40 mg/ml (p < 0.05). At PCW, all spirometry-parameters were markedly reduced: FVC by 41.3+/-16.4% (mean +/-SD); FEV_1_ by 44.7+/-14.5%; PEFR by 40.5+/-14.5 and FEF_25–75_ by 54.7+/-14.4% (P < 0.01 for all parameters). This reduction was accompanied by de-saturation, hyperpnoea, tachycardia and a response to bronchodilators.

**Conclusion:**

Determination of PC_20_-FEV_1_ by spirometry is feasible in many preschool children. PC_20_-FEV_1_ often appears at lower provocation dose than PCW. The lower dose may shorten the test and encourage participation. Significant decrease in spirometry indices at PCW suggests that PC_20_-FEV_1_ determination may be safer.

## Background

Measurements of bronchial hyper-reactivity (BHR) have provided insight into the physiological basis of asthma, and provide a tool for asthma diagnosis, assessment of asthma severity and response to treatment [1,2]. The bronchial provocation tests require an objective outcome measurement that reflects airway function. Forced expiratory volume in 1 second (FEV_1_) has been standardized to measure changes in airway caliber that occur with bronchial provocation [3]. In the Methacholine challenge test (MCT), the provocative concentration reducing FEV_1 _by 20% from baseline (PC_20_-FEV_1_) is considered the end point of the test. Traditionally, spirometry in young children has been difficult to achieve. Therefore, techniques that do not require cooperation (i.e., detection of wheeze during normal breathing, a fall of 5% in O_2_-saturation (SaO_2_), or an increase of 50% in respiratory rate and/or heart rate) have been used as alternative end points in bronchial provocation tests in the preschool age [4-7]. Recently it has been shown that young children can be taught to perform reliable forced expiratory maneuvers [8-11]. Yet, it is unclear whether these young children have the drive to perform and tolerate repetitive reproducible spirometry-sets that are measured during the interval between inhalations. Concentration of methacholine (MCH) causing wheeze, a fall of 5% in O_2_- Saturation, an increase of 50% in respiratory rate and/or heart rate (PCW) and PC_20_-FEV_1 _were compared in school children and a good correlation was found between the two methods [7,12-14].

This study assesses the ability of young asthmatic preschool children to cooperate with repetitive spirometry-sets during MCT, and thereby allow determination of PC_20_-FEV_1 _in comparison with PCW.

## Methods

### Subjects

Consecutive preschool children referred to the Pediatric Pulmonary Clinic, Meyer Children's Hospital, Rambam Medical Center, Haifa, over a 6-month period were recruited. Of 62 families offered participation in the study, seven refused. None of the children had experienced spirometry previously. *Inclusion criteria were*: 2.5–6.5 year-old children who were asthmatic according to GINA guidelines [15] with recurrent episodes of wheeze, cough and/or shortness of breath with clinical response to bronchodilator; normal chest auscultation and FEV_1 _>75% of predicted for healthy preschool children [9] after saline inhalation. *Exclusion criteria *were: presence of other chronic respiratory conditions; emergency room visit in the past three months; respiratory infection in the past month; oral or inhaled steroids or other anti-inflammatory medication taken in the last week; bronchodilator taken within 24 hours prior to the test.

The Rambam Medical Center Ethics Board approved the study. Parental consent was obtained for each child.

### Methacholine challenge

Tests were performed in a designated room at the Pediatric Pulmonary Unit, Meyer Children's Hospital, Haifa, Israel. A parent and the investigating team (a pediatric pulmonary physician, respiratory physiologist and technician) were present throughout the test. MCT was performed according to published guidelines, [3], with doubling doses of fresh Methacholine solutions (0.06 to 8.00 mg/ml) dissolved in saline. Solutions were driven by compressed air of 5 l/min flow (giving a mean output of 0.4 ml/min), and nebulized using a Hudson nebulizer (Hudson RCI, Temecula, CA, USA). Inhalations were performed using a facemask while the child was sitting up straight and breathing normally. Nebulized Methacholine was inhaled for 2 minutes, with 5-minute intervals between doses, until the maximal concentration or the end point was reached. To ensure safety in light of the risk of airway closer, the MCH increment was only half the usual amount when transient wheeze or cough was noted, keeping in mind that the accumulative dose is affected by this manipulation. Oxygen saturation and heart rate were monitored continuously by pulse oximetry (Biox 3700e; Ohmeda). A single observer (LB) performed auscultation for 20 seconds over the trachea and two zones of both lungs (upper front and lower back) according to Springer et al. [7]

The following indices were considered "end of test": appearance of audible wheeze, a fall of ≥5% in O_2_-saturation, or an increase of ≥50% in respiratory rate and/or heart rate [7]. At the "end of test", spirometric measurements were performed, followed by administration of nebulized Albuterol (2.5 mg).

### Spirometry

Forced expiratory flow volume (FEFV) curves were measured with a ZAN100 commercial spirometer (ZAN Messgeraete GmbH, Oberthulba, Germany). Calibration was performed before the testing sessions. The curves were monitored on the computer screen to ensure best effort. Results were corrected to BTPS conditions. The software included an interactive animated computer game (SpiroGame^®^) set by targets of the FEFV maneuver, combining forced inhalation preceding forced expiration, peak expiratory flow rate (PEFR) and forced vital capacity (FVC) with emphasis on prolonged expiration. [8] The targets were the extrapolated values derived from comparative data from older children, corrected for height. [16] An experienced pulmonary technician instructed each child how to operate the game. Teaching time was limited to 15 minutes. On-line rejection of curves was based on visual inspection for "non-cooperation" errors and included: poor effort; incomplete expiration; cough; glottis closure. Curves had to show a rapid rise to peak flow, and gradual, smooth decline of flow down to residual volume. Baseline maneuvers were repeated to visually obtain best possible efforts on at least 3 technically acceptable FEFV curves. After obtaining baseline spirometry, MCT was performed. A duplicate spirometry set was performed immediately after auscultation. PC_20_-FEV_1 _was determined off line by the provocative concentration that reduced FEV_1 _by 20% from baseline. PC values were log-transformed before statistical analyses. Spirometry indices included FVC, FEV_1_, PEFR, forced expiratory flow at 50% FVC (FEF_50_), FEV_1_/FVC ratio.

### Analysis and statistics

Three baseline spirometry curves were analyzed for acceptability criteria according to ATS/ERS guidelines [17,18] and in comparison with similar data for preschool children [11,19]. These included: a) "Start of test" criteria: time to peak expiratory flow and backward extrapolated volume (Vbe) b) "End of test criteria": described by "total expiratory time" and the ratio of "no change in expiratory volume" to "total expiratory time" c) reproducibility (coefficient of variation) of the three baseline curves, calculated as SD/mean*100.

After inhalations, the curves were inspected visually online, and were analyzed offline in relation to baseline using paired t-test. Differences were considered significant when p < 0.05. The level of agreement between the dose at end of test and the dose of PC_20_ were compared by Bland and Altman analysis (20).

## Results

A total of 55 children (28F/27M, age range 2.8–6.4 years) were recruited. Eleven children failed spirometry and underwent MCT by auscultation only. Failure to perform spirometry was due to lack of comprehension (4 children) or failure to repeat spirometry after baseline measurements (7 children). Failure was not age dependent. Eight children refused to cooperate with either test. Thirty-six of 55 (65.5%) children performed the MCT with spirometry tests and with auscultation. Of these 36 children, eleven were 2.5–3.9 years old, 15 were 4–5 years old, and 10 were >5 years old. Three children failed to produce FEV_1 _on the baseline measurements but were able to produce it after saline administration. In these children, post saline FEV_1 _measurements were considered as baseline. FEV_1 _at that point was >75% predicted. The anthropometric data and baseline lung function of the 36 patients are presented in Table 1 and clinical characteristics in Table 2.

**Table 1:** Anthropometric data and lung function. The results are expressed as mean ± SD.

Anthropometric data				Baseline lung function %predicted [16]				
N	Height (cm)	Weight (kg)	Sex (M/F)	FVC	FEV1	FEV1/FVC	PEFR	FEF_50_
36	104 ± 7	18 ± 3	20/16	95 ± 15	91 ± 14	96 ± 3	99 ± 14	101 ± 16

**Table 2:** Clinical Characteristics

N = 36	Recurrent cough	Recurrent lung infiltrates	Shortness of breath	Wheezing	Atopy	Family history of allergy
N	35	24	24	16	16	23

The 36 children participating in both tests had a previous response to bronchodilators as judged by clinical observation. The average duration of respiratory symptoms was 18 ± 14 weeks. Five children were not receiving any medication for a period of weeks. Nine children were receiving bronchodilators as needed, and 22 were using both inhaled steroids and bronchodilators as needed.

Quality of baseline maneuvers: Start of test: Peak expiratory flow rates were reached within a mean of 98 ± 7 ms (range 89–115 ms) and mean Vbe was 3.4 ± 1.5% of FVC (range 1.2–5.7). Intra-subject reproducibility for the baseline triple maneuvers was: for FVC, 4.1 ± 2.3% (range 1.8–6.3); for FEV_1_, 3.8 ± 2.3% (range 0.4–7.3); for PEFR, 4.4 ± 2.8% (range 0.3–8.6) and for FEF_25–75_, 7.9 ± 3.5% (range 2.7–13.2). *End of test: *Mean expiratory time was 1.48 ± 0.47 seconds and the ratio of "no change in expiratory-volume" to "total expiration time" was 0.20 ± 0.06.

### MCT test

**Figure 1:**
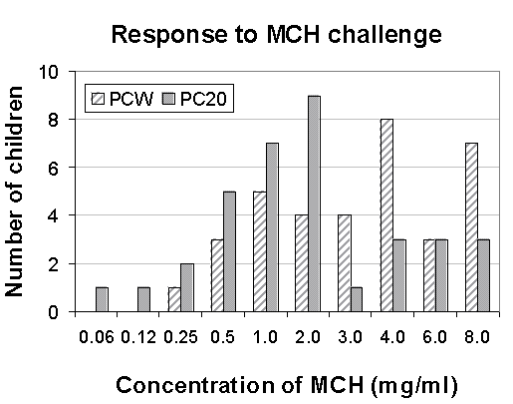
Number of children responding to each MCH concentration (mg/ml) at PCW and at PC_20_-FEV_1_

Children's response to MCT (n = 36) is summarized in Figure 1 and Table 3. Average test time to reach PC_20_-FEV_1 _was 29 ± 11 minutes, while for PCW it was 41 ± 10 minutes (not including bronchodilator administration) (p < 0.001). The end point of the challenge was determined by the pediatric pulmonologist as positive in 35/36 children. One child did not display any of the determined criteria for PCW up to 8 mg/ml and was considered to have no BHR. The mean (± SD) concentration at PCW for the 36 children was 4.26± 3.31 mg/ml. Wheezing at the end point was observed in 26/36 children and in 9/36 the test was ended before the appearance of wheeze due to either oxygen desaturation or tachypnea accompanied by audible long expiration. Mean increase in heart rate at PCW was 25.5 ± 11% (range 10–42%); respiratory rate increased by 30.0 ± 21.1% (range 0–42%) and SaO_2 _decreased by 6.3 ± 2.7% (range 2.3–10.3%).

**Table 3:** Appearance of respiratory distress signs at PCW and PC_20_-FEV_1_

Symptom	Cough	Wheeze	Prolonged Audible Expiration	Decrease SaO_2_	Increased HR	Increased RR
# Children at PCW	32	26	24	33	28	25
# Children at PC_20_-FEV_1_	28	2	7	15	3	7

**Figure 2:**
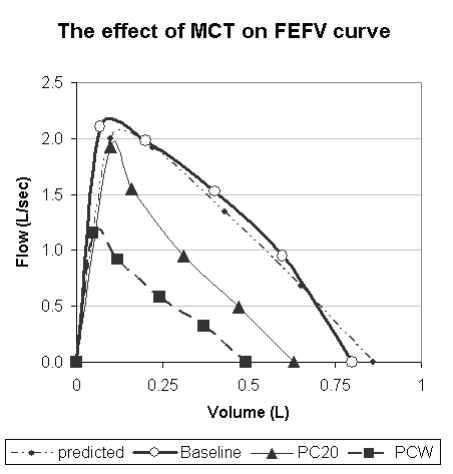
A representative example of forced expiratory flow-volume curves from one child. Predicted, Baseline, PC_20_-FEV_1 _and PCW curves are presented

PC_20_-FEV_1 _occurred at a mean concentration value of 1.96- ± 1.83 mg/ml. The one child who did not respond to MCH of up to 8 mg/ml by PCW (negative BHR) did not show a fall of 20% from baseline FEV_1 _value either. The other 35 children exhibited a fall of 20% in FEV_1 _from baseline values in response to MCH ≤8 mg/ml (Figure 1 and Table 3). A representative set of FEFV curves from a single patient that includes the predicted curve, baseline, PC_20_-FEV_1 _and end of test curves is shown in Figure 2.

**Figure 3:**
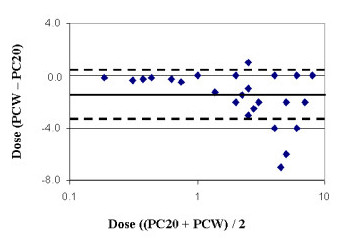
Analysis of the difference in dose values at end of test (PCW) and the dose at PC_20_, as compared with mean Dose values of the two, in a Bland and Altman analysis (20). Dotted lines represent 95% coefficient of variation values.

At PC_20_-FEV_1 _there was a mean increase in heart rate of 13.5 ± 11.0%, respiratory rate increased by 15.4 ± 15.8% and SaO_2 _decreased by 2.4 ± 2.1% from baseline level. These changes were significantly lower than those found at PCW (p < 0.01 for three parameters). The appearance of PC_20_-FEV_1 _occurred 2 concentrations earlier than PCW in 5 children, 1.5-concentrations earlier in 3 children, one concentration earlier in 17 children, 0.5 concentrations earlier in 3 children and at the same concentration as PCW in 7 children (Figure 1). The effects of MCH on the spirometry parameters are presented in Table 4. At PC_20_-FEV_1_, parameters were moderately decreased, while at end point, test parameters were markedly reduced. The severity of FEV_1 _reduction at PCW was variable, ranging from 30.8 to 68.2% of baseline. The level of agreement between the dose at end of test (PCW) and the dose at PC_20_[20] is presented in Figure 3. *Dotted lines *represent 95% coefficient of variation values.

**Table 4:** Changes in respiratory indices at PCW and at PC_20_-FEV_1_. The results are expressed as mean ± SD. (n = 35/36, as one child did not respond to MCH and his spirometry did not change throughout the test).

Parameter	End of test	PC_20_-FEV_1_
FVC	- 41.3 ± 15.5	- 18.4 ± 10.0 *
FEV_1_	- 44.7 ± 14.5	- 24.6 ± 6.4 *
FEV_1_/FVC	- 6.09 ± 6.8	- 4.1 ± 3.8 *
PEFR	- 44.2 ± 13.2	- 21.4 ± 10.6 *
FEF_50_	- 61.2 ± 14.2	- 38.6 ± 16.9 *
Expiratory time (sec)	+2.8 ± 0.4	+2.2 ± 0.4 *

* Changes at PC_20_-FEV_1_ are significantly lower than at "end of test", p < 0.01

Bronchodilators improved FEV_1 _by 43 ± 29% from PCW values and all respiratory symptoms disappeared shortly after bronchodilator administration.

## Discussion

In this study we assessed the feasibility of determining PC_20_-FEV_1 _during Methacholine bronchial provocation testing in asthmatic preschool children. We found MCT was feasible in 65% of this group of wheezy preschool children. Children as young as 3 years old complied and cooperated with what seems to be a most fatiguing procedure. Baseline measurements met most of the ATS criteria for older children and adults [17,18] and quality control studies on spirometry in preschool children [11,19]. We found that PC_20_-FEV_1 _correlates with PCW. However, PC_20_-FEV_1 _frequently precedes PCW. All spirometry parameters at PC_20_-FEV_1 _were significantly higher than those measured at PCW.

In this study, we used interactive spirometry games [8] with multiple spirometry targets, since single targeted games (usually peak expiratory flow targeted) have not fulfilled expectations [21,22]. Our teaching method is supported by the findings that 65% of the children fully cooperated not only with baseline measurements but also with spirometry sets. Of note, 26 of the 36 children were younger than 5 years. Conforming quality control was necessary to proceed with the test. The quality control of baseline spirometry in our study met most ATS/ERS criteria concerning reproducibility and start of test criteria [17,18] and matched those reported for preschool children [11,19], encouraging us to continue with the MCT test. Vbe = 5%FVC found in our study is narrower than reported [11], as we have rejected in advance curves with Vbe >5%FVC at the expense of success rate. It should be stressed that our work did not compare verbal coaching [9] or other spirometry games [11,23] as the preferable methodology for keeping the child going and performing repetitive spirometry sets.

The mean PC_20_-FEV_1 _of 1.96 ± 1.83 mg/ml found in our group reflects a mild degree of BHR, as we recruited children with mild asthmatic symptoms. Our findings for PC_20_-FEV_1 _are comparable to those of Hayden et al [13], who found a mean PC_20_-FEV_1 _at FEV_0.5 _of 2.49 ± 2.55 mg/ml in infants. Adinoff et al [24] reported a mean provocative dose of 3.0 mg/ml Methacholine in their preschool children and infants. Tepper [25] et al. reported that infants with asthma-like respiratory symptoms might respond to MCH concentrations as low as 1.25 mg/ml. In that respect we found that PC_20_-FEV_0.5 _occurred at a mean concentration value of 1.29- ± 1.47 mg/ml, meaning that the responsiveness of the airways in the preschool age may be similar to that of infants, despite differences in the measurement techniques. It is important to note that PC_20_-FEV_0.5 _occurred at a significant mean lower concentration than PC_20_-FEV_1 _(1.96- ± 1.83 mg/ml; p < 0.01), however, standardization is needed to accept the PC_20_-FEV_0.5 _value for the determination of hyper-reactive airways.

### PCW

We found that PCW occurred in our group at a mean concentration of 4.26 ± 3.31 mg/ml. PCW values in our study were much higher than the PCW (0.4 mg/ml) reported by Springer et al [7]. The difference may be attributed to inclusion of more severe asthmatics in their study group.

### Spirometry at PCW

We found that PC_20_-FEV_1 _occurred at a lower concentration than PCW in most subjects. This finding is in agreement with several other studies comparing PCW detection to PC_20_-FEV_1 _in school age children. [4-7,14]. However, in none of these studies were spirometry measurements carried out to the point of wheeze. We expected to find a good correlation between the two tests (PCW = 1.2195*PC_20_-FEV_1 _+ 0.0288; R^2 ^= 0.9733; p < 0.005), yet the Blant and Altman analysis revealed that in children with higher mean provocation PCW dose (≥6 mg), the level of agreement between the methods was low, reflecting higher sensitivity of the PC_20_ method, especially in mild airway reactivity (Figure 3).

**Figure 4:**
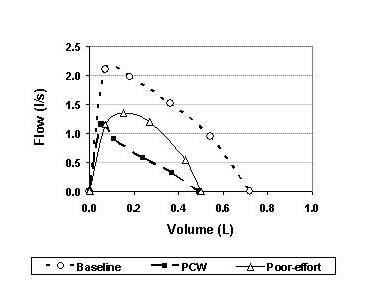
A representative example of poor-effort forced expiratory flow-volume curves from one child. Baseline, Post challenge and poor effort during teaching process are presented.

We found that at PCW, FEFV curves visually seemed to be smaller and all parameters were reduced simultaneously (Figure 2), with a highly significant reduction in flows and volume parameters. The reduction in curve was gradual in most children, accompanied by an increase in respiratory symptoms (Table 2), and responded to bronchodilators, and hence was not considered to reflect fatigue. To further strengthen this point a representative curve of one child illustrating, a poor effort performed at teaching process vs. end of test curve is shown in figure 4. The poor-effort curve did not fulfill start of test criteria and is round while the "end of test curve" has an obstructed shape.

The reduced FVC and flows are most likely due to a severe degree of airway narrowing involving small to medium airways that may be accompanied by air trapping, partial closure of airways and elevation in FRC. Reduced FVC may also be due to increased glottic narrowing due to MCH irritation [26,27], but the flow volume curve was not suggestive of upper airway obstruction (trimmed PEFR). Alternatively, the upper airways response to methacholine may contribute to the increase in total respiratory resistance [27]. This pattern occurred in some cases before appearance of wheeze or other clinical end-points. Indeed, in 9/36 subjects, the test was terminated due to oxygen desaturation or tachypnea rather than wheeze. Similar to our results, Sprikkelman et al [28] reported that wheeze was detected in only 33% of 15 school-age asthmatic children at PC_20_-FEV_1, _and Springer et al [7] terminated the test without the presence of wheeze in 19.2% of young children. In this respect we would argue that FEV1 does make a contribution beyond simply asking the subject if they wheeze. Novitzki et al (4) found in 5–8 year-old children that FEV_1 _is decreased by 33.3 ± 7.4% at PCW. Spence et al [29] reported a mean fall of 51 ± 14% from baseline FEV_1 _when wheeze appeared in their asthmatic older subjects. Our results strengthen these prior findings, and suggest that spirometric PC_20_-FEV_1 _may be achieved with inhalation of lower MCH concentrations than those used to achieve wheeze.

Measuring PCW during tidal volume breathing has the advantage that no active cooperation on the child's part is needed. Therefore the success rate of PCW is higher than spirometry (44/55 children). However, using PC_20_-FEV_1 _(or PC_20_-FEV_0.5_) can preclude inhalation of higher concentrations of MCH used to achieve wheeze, leading to alarmingly diminished flows found at PCW and a significant shortening of test time relative to PCW.

## Conclusion

We conclude that PC_20_-FEV_1 _is feasible in preschool asthmatic children when using respiratory games teaching techniques and that the children tolerate repetitive duplicate sets of spirometry maneuvers. PC_20_-FEV_1 _in preschool children appears to be as sensitive as in adults and school children. Yet, many questions remain open as to the usefulness of this test in a random sample of young children and/or how discriminating this test is as a diagnostic tool. It would also be necessary to assess the sensitivity of this test to various severities of disease. Further studies are needed for standardization and definition of methodological criteria.

## Competing interests

The author(s) declare that they have no competing interest.

The SpiroGame program is privately patented in USA, granted to Dr. Vilozni.

Dr. Vilozni does not foresee any financial gain or loss, now or in the future from publishing this manuscript. The patent is not commercialized.

## Authors' contributions

Dr. Lea Bentur and Dr. Daphna Vilozni had primary responsibility for protocol development, outcome assessment, data analysis and writing of the manuscript.

Dr. Raphael Beck, Dr. Nael Elias, Dr. Asher Barak, Dr. Ori Efrati and Prof. Yaacov Yahav contributed to this study by patients screening, patient enrollment, analysis of the data and quality control of the data.

## Pre-publication history

The pre-publication history for this paper can be accessed here:


